# New tools for diagnosis of primary immunodeficiencies: from awareness to artificial intelligence

**DOI:** 10.3389/fimmu.2025.1593897

**Published:** 2025-07-10

**Authors:** Pere Soler-Palacín, Jacques G. Rivière, Siobhán O. Burns, Nicholas L. Rider

**Affiliations:** ^1^ Department of Pediatrics, Obstetrics and Gynecology, Preventive Medicine and Public Health, Universitat Autònoma de Barcelona, Barcelona, Catalonia, Spain; ^2^ Infection and Immunity in Pediatric Patients Research Group, Vall d’Hebron Institute de Recerca (VHIR), Barcelona, Catalonia, Spain; ^3^ Pediatric Infectious Diseases and Immunodeficiencies Unit, Hospital Infantil I de la Dona Vall d’Hebron, Vall d’Hebron Barcelona Hospital Campus, Barcelona, Catalonia, Spain; ^4^ Jeffrey Modell Diagnostic and Research Center for Primary Immunodeficiencies, Barcelona, Catalonia, Spain; ^5^ Institute for Immunity and Transplantation, University College London, London, United Kingdom; ^6^ Department of Clinical Immunology, Royal Free London National Health Service (NHS) Foundation Trust, London, United Kingdom; ^7^ Virginia Tech Carilion School of Medicine, Department of Health Systems and Implementation Science, Roanoke, VA, United States

**Keywords:** inborn errors of immunity, diagnostic delay, screening, artificial intelligence, machine learning, primary immunodeficiency

## Abstract

Primary immune deficiencies (PI) are rare diseases associated with frequent, severe infections, inflammatory and autoimmune diseases and/or cancer. Because of the variability in presentation, undiagnosed PI patients can be encountered by many different medical specialists. A lack of awareness of and the rarity of PI can lead to delayed diagnosis particularly among primary care physicians and non-immunology specialists. These delays can lead to irreversible sequelae, decreased quality of life and premature mortality. In this review, we describe two projects designed to decrease the time to diagnosis in PI patients: 1) the expert-driven PIDCAP project conducted in Spain to promote early diagnosis in the primary care setting, and 2) a multi-modal data-driven approach using artificial intelligence and machine learning to identify individuals at high risk for PI. Both approaches aim to create widely available tools to promote early diagnosis and treatment of PI. Initial results have been positive. Future directions include larger studies and potentially combining expert-driven and data-driven approaches.

## 1 Introduction

Inborn errors of immunity (IEI) are a heterogenous group of around 500 rare diseases that can present with a number of different clinical features including severe and recurrent or opportunistic infections, inflammation, autoimmunity and/or malignancy. Congenital immune defects with increased susceptibility to infection along with similar immune system dysfunctions that do not have a defined genetic basis, make up primary immune deficiencies (PI). Due to the multisystem nature of PI, patients can present to multiple medical specialties (1–3). Lack of awareness among primary care physicians and non-immunologists and the rarity of PI means that diagnosis is commonly delayed, often for many years (1, 4, 5). In addition, it is estimated that up to 70% of patients with PI may remain undiagnosed (6). Late diagnosis results in delayed treatment with accumulation of co-morbidities including irreversible end-organ damage.

## 2 The importance of early diagnosis of PI

**Figure 1:**
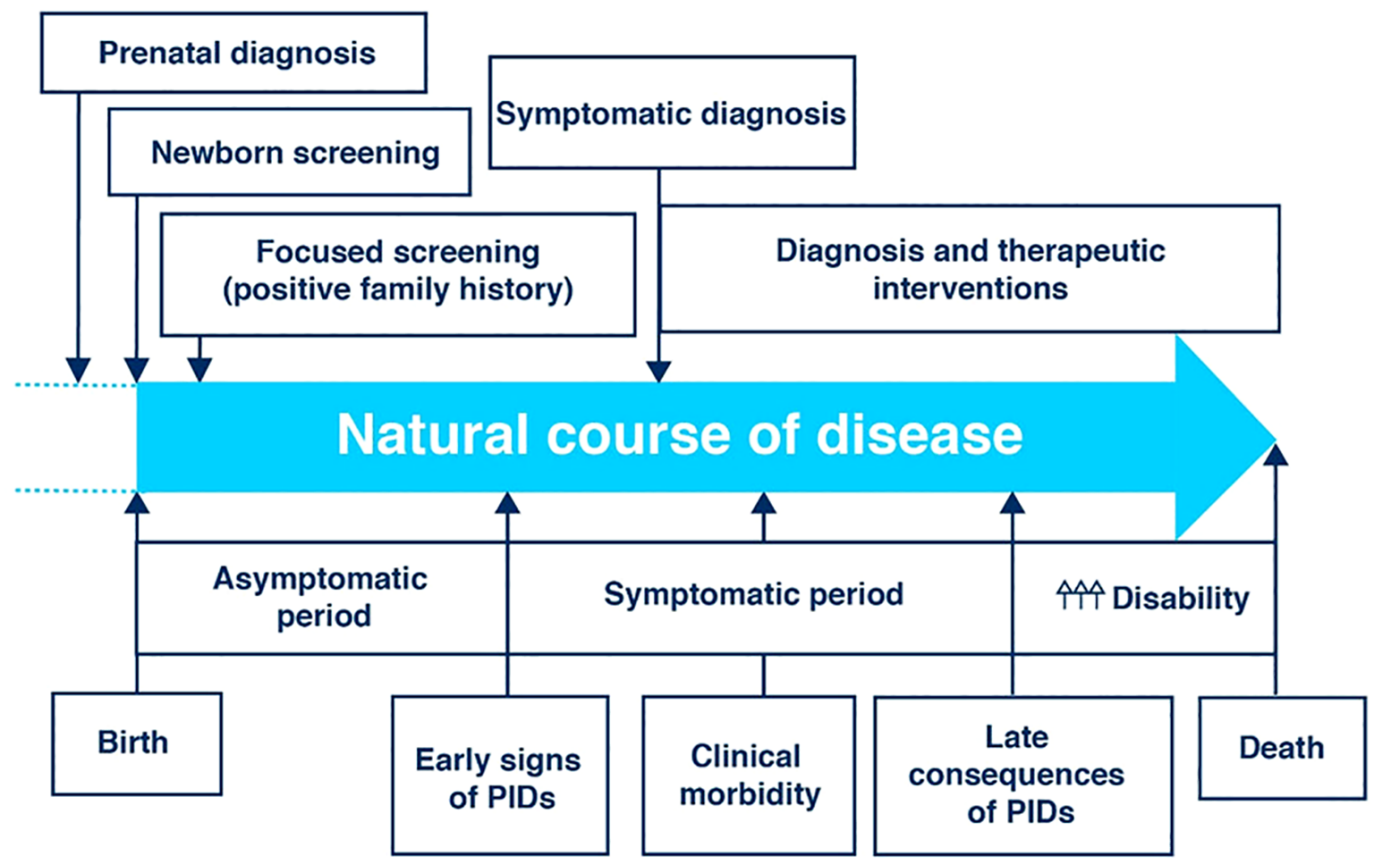
The clinical course of primary immunodeficiency (PID) showing the potential points of early diagnosis and intervention [Adapted from Jesenek et al. (10)]. Licensed under CC-BY.

Early diagnosis is a key factor in limiting the impact of PI on patients’ lives (**Figure 1**). Earlier diagnosis and treatment have been shown to increase quality of life and survival in PI patients while decreasing the overall financial burden on patient and the healthcare system (7). The goal of some early diagnostic interventions, such as newborn screening programs, is to diagnose patients with PI during the asymptomatic phase of their disease. For many types of PI, pre-symptomatic diagnosis is currently possible, in which case diagnosis as early as feasible in the symptomatic period could still allow avoidance or delay of the most serious sequalae.

Therefore, an important focus for improving outcomes is maximizing diagnostic opportunities in the PI disease journey for these patients. There are four major potential diagnostic opportunities in this journey: 1) newborn screening, 2) family history and early symptomatic phase in primary care, 3) specialized non-immunological secondary care, and 4) specialized referral units. Diagnosis can take place early in the disease process (prenatal or newborn diagnoses or screening based on family history) or later in the disease process after symptoms have appeared (symptomatic diagnoses). Contributing to the complexity of PI diagnosis is the number of potential genetic defects underlying the condition. (**Figure 1**).

## 3 Newborn screening programs

As noted above, newborn screening programs are the first opportunity for diagnosis in the journey of patients with IEI. One such newborn screening program is testing for severe combined immune deficiency (SCID). A number of countries have nationwide newborn screening programs (Canada, Czech Republic, Denmark, Finland, Germany, Iceland, Ireland, Israel, Japan, The Netherlands, New Zealand, Norway, Sweden, Switzerland and the United States) and additional countries have regional programs (Australia, Italy, Spain and Vietnam). Still others are actively conducting pilot projects (France and the United Kingdom), but the majority of countries do not currently have screening programs for SCID (8).

Newborn screening programs are evolving to apply next generation sequence and can potentially include whole genome sequencing (8). An example of the potential for whole genome sequencing is the GUARDIAN study. Interim results from this study suggest that genome sequencing can improve screening for conditions already detected by current newborn screening and expand screening to conditions not amenable to current testing techniques (9).

The rate of discovery of single-gene defects underlying PI has expanded rapidly in recent years, with the total number of genes identified exceeding 555 in 2024 (11–13). These defects span 10 different categories of IEI (**Table 1**). This diversity creates diagnostic challenges due to the sheer number of potential underlying genetic defects and the heterogeneity and variable genetic penetrance in the way these disorders can present.

**Table 1:** Categories of IEI and related genes (from Tangye et al. [11)].

Categories of IEI	Number of Genes Identified	Genes Involved in Recently Identified Defects
Combined Immunodeficiencies	66	*LCP2, PAX1, ITPKB, SASH3, MAN2B2, COPG1, IKZF2, CHUK, IKZF3, CRACR2A, CD28*
Combined Immunodeficiencies with Syndromic Features	69	*MCM10, IL6ST, DIAPH1*
Predominantly Antibody Deficiencies	45	*FNIP1, SP11, PIK3CG, POU2AF1, CTNNBL1, TNSRSF13*
Diseased of Immune Dysregulation	52	*RHOG, SOCS1, PDCD1, ELF4, TET2, CEBPE, IKZF1*
Congenital defects of phagocytes	42	*CXCR2*
Defects in Intrinsic and Innate Immunity	74	*TBX21, IFNG, TLR8, NOS2, SNORA31, ATG4A, MAP1LC3B2, ZNFX1 TLR7, MAPK8*
Autoinflammatory Diseases	56	*TMEM173, LSM11, RNU7-1, CDC42, STAT2, ATAD3A, TBK1, RIPK1, NCKAP1L, SYK, HCK1, IKBKG, PSMB9*
Complement Deficiencies	36	None recently identified
Bone Marrow Failure	44	*MECOM1*
Phenocopies of IEI	14	*TLR8*

Licensed under CC-BY 4.0.

The expansion of genomics, while increasing diagnostic reach, raises important ethical and economic considerations. Ethically, the identification of variants of uncertain significance, gene defects with unclear genotype-phenotype correlations, or conditions lacking established therapeutic interventions may lead to uncertainty and anxiety for families. Economically, although sequencing costs have declined, the scalability and sustainability of genome-wide screening across broad populations remain under evaluation. Programs such as the GUARDIAN project (guardian-study.org) and the Generation Study (Newborn Genomes Programme | Genomics England) will be critical to determine the real-world feasibility, cost-effectiveness, and clinical impact of integrating genomic technologies into routine newborn screening.

## 4 Diagnosis in the primary care setting

Especially if it is not recognized or tested for in newborn screening, the challenges of recognizing PI can lead to diagnostic delays that have a median of 4-9.5 years but can extend to decades (14–17). The diagnostic delay can vary somewhat depending on the underlying immune deficiency. For common variable immunodeficiency the diagnostic delay has been estimated at 8.8 years (95% confidence interval 8.2-9.3 years) (14), for immune dysregulatory disease 5 years (interquartile range 1–14 years) (15), for monogenic inborn errors of immunity 3.3 years (17), and for primary antibody deficiencies 9.5 years (16). Numerous factors contribute to these long delays including, the diversity of symptoms and clinical features at presentation, inappropriate referrals and lack of consideration of a rare disease (17, 18).

Regardless of the reasons for delayed diagnoses in PI patients, detrimental outcomes from the delay can be significant. Diagnostic delays can lead to progressive organ damage from repeated infections and excessive costs due to non-productive referrals and ineffective or temporarily effective treatments (14, 16, 17). Currently 49% of all PI patients have some degree of permanent functional impairment at the time of diagnosis (19). Given the damage that a delayed diagnosis can cause, data science and new technologies can be used to shorten the diagnostic odyssey for PI patient. Diagnostic delays in the primary care setting can result from a lack of awareness of PI among other factors.

## 5 Promoting PI diagnosis in primary care

To aid in the diagnosis of PI, especially in the primary care setting, ‘The 10 warning signs of PI’ were proposed by the Jeffrey Modell Foundation in conjunction with their medical advisory board and the American Red Cross in 1993 (20). Currently there are pediatric and adult versions of these warning signs. They have been the basis for increased awareness in the years since their introduction and have been the key to increased referrals and earlier diagnosis. They have stood the test of time but were based primarily on infectious symptoms. They evolved forming the underpinnings of the Jeffrey Modell Foundation SPIRIT Analyzer (21) and have proven highly useful for population-wide screening of PI risk (22, 23).

Yet given the expanded understanding of new PI phenotypes which include autoinflammatory disease, immune dysregulation, autoimmune diseases or malignancies (11) a reappraisal of the 10 Warning Signs for PI is warranted. This hypothesis has been experimentally shown in an analysis of a very large US sample (n=73M) where features of autoinflammation and immune dysregulation were incorporated into the Warning Signs (24). Prior to the latter study, others have considered amending the 10 Warning Signs (2, 25–27) and new approaches considered for improving the early diagnosis of PI. In this article, two examples of different types of models for utilizing new techniques to improve diagnosis will be discussed: an expert-driven model and a data-driven model.

## 6 A new expert-driven approach: the PIDCAP project

**Figure 2:**
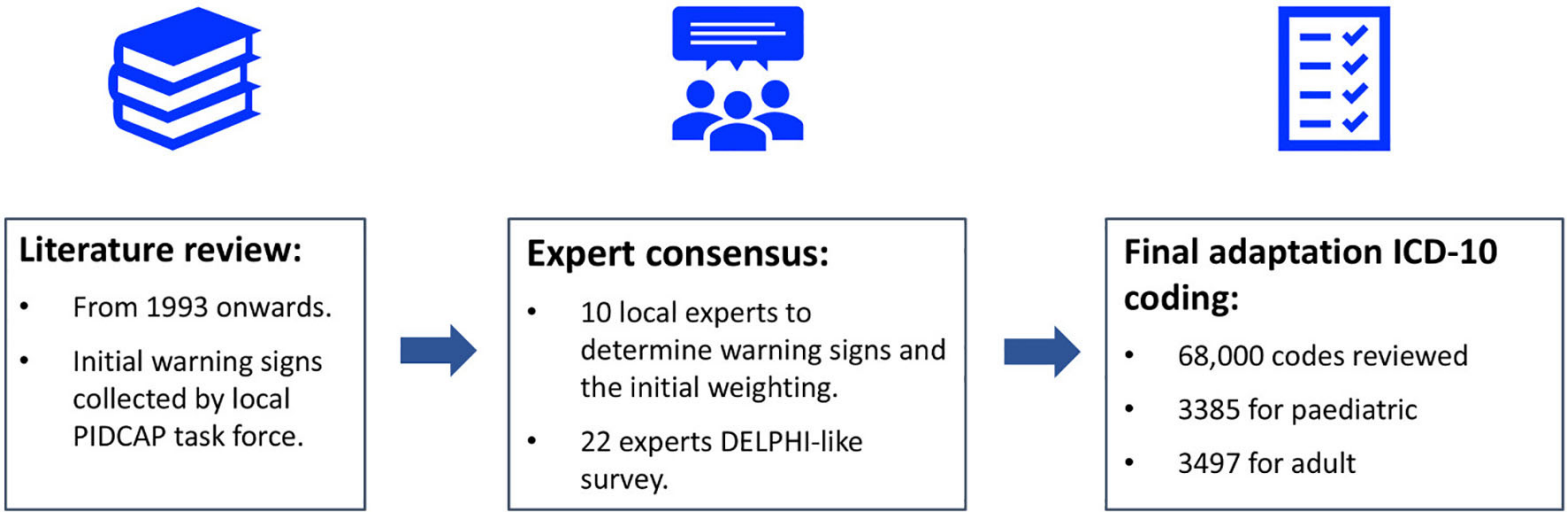
Algorithm for re-evaluation of PI warning signs by the PIDCAP Project.

The process of assigning weight to various diagnostic parameters can be done computationally or by using panels of experts. The PIDCAP project from Vall d’ Hebron Barcelona Hospital Campus was developed to update and adapt the PI warning signs for primary care, to help in the identification of individuals at high risk for PI and ultimately to shorten the time between initial presentation of the patient to the primary care physician to referral to the specialty care unit (**Figure 2**) (28). Developed in the Catalan Health Care System in Catalonia, Spain, this was an expert-driven model that assigned weights to warnings signs based on ICD-10 codes. The model started with a literature review (from 1993 to present) and the generation of an initial list of warning signs from the local PIDCAP task force. This list was reviewed by an expert consensus panel comprised of 10 Catalan and Spanish experts attending pediatric and or adult PI patients (n=6) along with primary care general practitioners and pediatricians (n=4) resulting in a list of warning signs with an initial weighting given to each sign. The expanded list of warnings was assessed by 22 experts throughout Spain in a DELPHI-like survey. This group narrowed the final set of warning signs to a weighted list of 26 signs for pediatric patients and 22 for adult patients. From the total of 68,000 possible ICD-10 Codes reviewed, 3,385 and 3,497 codes were selected for pediatric and adult patients, respectively, and assigned to the corresponding warning signs (**Figure 2**).

**Figure 3:**
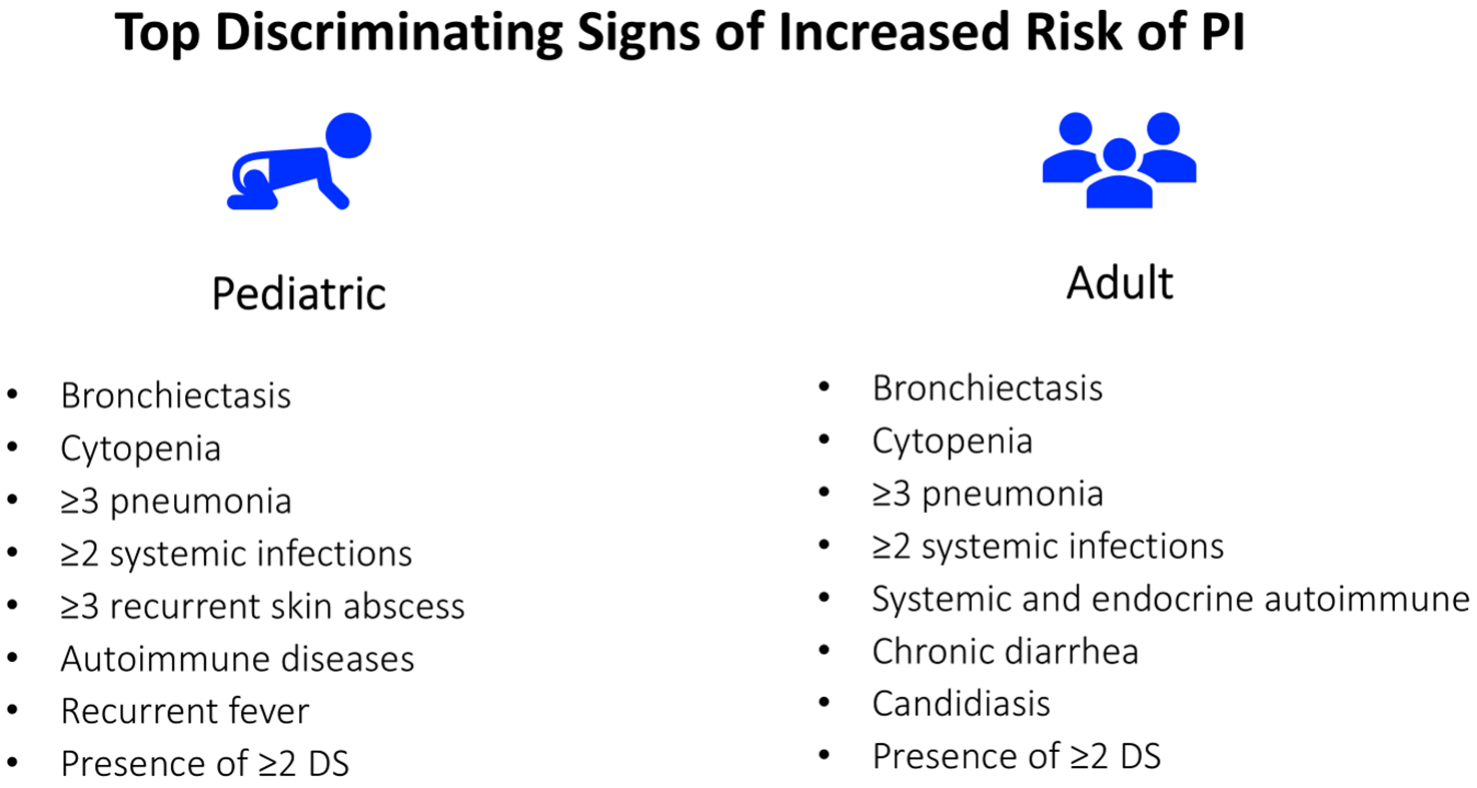
The top discriminating signs (DS) of increased risk for PI in pediatric and adult patients identified in the initial phase of the PIDCAP Project.

The system was integrated into electronic health software to alert primary care professionals of the PI risk score for individual patients. The pilot initiative was implemented in real-world practice in 2019 in the Catalan Health Care System to test the feasibility and implementation, specifically testing coordination between the primary and tertiary health care systems. The pilot area encompassed approximately 100,000 individuals (28, 29). High risk individuals were detected with a rate of 0.5% in pediatrics and 1.7% in adults. Overall results were positive with new cases of PI detected but the project was put on hold due to Covid-19 pandemic. Bronchiectasis, autoimmune disease, cytopenia, and recurrent infections were the most distinguishing warning signs for both adults and children (**Figure 3**). The integration of warning signs beyond classical infections was particularly important in the adult population, where common variable immunodeficiency may present solely with autoimmune manifestations. The scoring system was tested on a cohort previously diagnosed with PI at Vall d’Hebron Hospital detecting 32% and 30% of pediatric and adult cases, respectively. What could appear to be an important limitation of this initiative, that is a rather low detection percentage, was purposefully designed to avoid alert fatigue in primary care and a high percentage of false positives as the main goal was to increase awareness with a plan to fine tune the process in the future.

These results will help with refining the weighting of warning signs when they are tested on broader populations. Toward that end, an international DELPHI survey has been initiated to reach a broader consensus and the testing of the PIDCAP system in other populations and countries is planned.

In the PIDCAP project, primary care physicians with children at high risk for PI were notified to follow up with the patients. These physicians were presented with three courses of action: 1) order an immunological work-up which included full blood counts and Ig levels and determine further action based on those results; 2) provide the patient/caregivers with a virtual referral to a specialized treatment center; or 3) set up a referral and in person visit to the specialized treatment center.

There are benefits and drawbacks to patients receiving de-centralized care at their local hospital versus centralized care at a referral hospital (**Table 2**). Local hospitals offer advantages in reduced indirect costs to the patient (travel, hotel, meals, etc.) and cause less disruption to the patient’s quality of life. Unfortunately, limitations in resources and experience at local hospitals may be more likely to result in a delay in diagnosis. On the other hand, centralized care at referral centers provides a high level of expertise often along with cutting edge research, specialized laboratory immunology tests and participation in patient registries.

**Table 2:** Advantages and Disadvantages of Centralized and De-Centralized Care for PI.

	De-centralized Care @ Local Hospitals	Centralized Care @ Referral Centers
Available Specialists	+/-	++
International Network	–	++
Patient QoL	++	+/-
Clinical Trial Participation	+/-	++
Participation in Patient Registries	+/-	++
Advanced Lab Tests, Off-Label Medication Use	+/-	++
Environmental Benefits/Lower Indirect Costs	++	+/-
Diagnostic Delay	++	+/-

In addition, referral hospitals can help with the diagnosis and treatment of patients with PI through advanced genetic testing. With newer genetic testing technologies, the capacity of sequencing systems has increased exponentially (30). In addition, sequencing has become faster and more affordable and has gained wider use in common clinical practice. The advances in sequencing technology present new challenges as well and among these are: choice of the appropriate sequencing technology, interpretation of the data and management and storage of data. Many of the less common and more in-depth sequencing techniques and downstream functional validation tests are only available through research laboratories at referral centers and help local hospitals to clarify diagnoses in patients where the routine genetic tests do not provide definitive results.

A mixed treatment model bringing together patients and providers from both local and referral hospitals could potentially provide better and more efficient treatment of PI. Utilizing the convenience and lower cost of local hospitals with the advanced diagnostics and care at referral hospitals could improve care for patients with PI and provide better use of healthcare resources.

## 7 Utilizing professional networks to enhance PI diagnosis and management

Another set of tools that can help provide the most up-to-date diagnostics and treatment for patients with PI are professional networks. They can also help provide a connection between local hospitals and referral centers which can potentially help shorten the patient’s journey from symptom onset to effective diagnosis and treatment. Professional networks are also of extreme importance in helping improve detection and treatment at national and international levels. Patient registries, scientific societies, rare disease foundations and patients’ associations can all play a role. In Europe, the ERN-RITA Network is an important organization for immunodeficiency and other immune disorders (31). They provide disease information on PI, virtual consultations and a list of referral centers throughout Europe. Through organizations like ERN-RITA, specialist physicians at the referral centers can help guide the treatment of patients at distant hospitals. For many of these patients, frequent travel to the referral center would be time-consuming and costly.

Also, other private initiatives facilitate the connection between health care providers and PI specialists, ensuring that patients can obtain access to the latest research and best practices. Examples of these initiatives include the Immune Deficiency Foundation’s “Consulting Immunologist Program,” the Jeffrey Modell Foundation’s Global Centers Network which facilitates expert referral and patient navigation, and the Clinical Immunology Society’s PIDD listserv, an international platform for real-time peer consultation among immunologists. Through these networks newer tools and treatment alternatives can be available to healthcare providers at smaller hospitals.

One of the ways that physicians at smaller hospitals in Europe can tap into the expertise at referral centers is through the ERN Clinical Patient Management System (CPMS) (32). In the CPMS, after obtaining the patient’s consent, they can be enrolled and experts across the ERN system are invited to a panel discussion of the patient’s case. At the conclusion of the discussion, a final report is issued to help guide the patient’s diagnosis and treatment. This process can be time-consuming, but the CPMS Working Group and ERN-RITA have been working to make it more streamlined.

Another example of how experts at referral centers can help physicians at local hospitals is the Clinical Immunology Society (CIS) Primary Immunodeficiency Diseases (PIDD) listserv (33). In this forum, immunologists and researchers can post questions and exchange information regarding the investigation and treatment of PI. While this is a much more informal system than the CPMS, it still promotes collaboration and information exchange in the interest of improving care for individuals with PI.

Networks such as the ERN CPMS and the CIS PIDD listserv can provide critical information to aid primary care physicians in diagnosing and treating patients with PI. In addition, these networks can provide a bridge between local hospitals and referral centers for consultation and/or patient transfer based on the needs of the individual patient.

## 8 Future directions: using artificial intelligence to improve PI diagnosis

**Figure 4:**
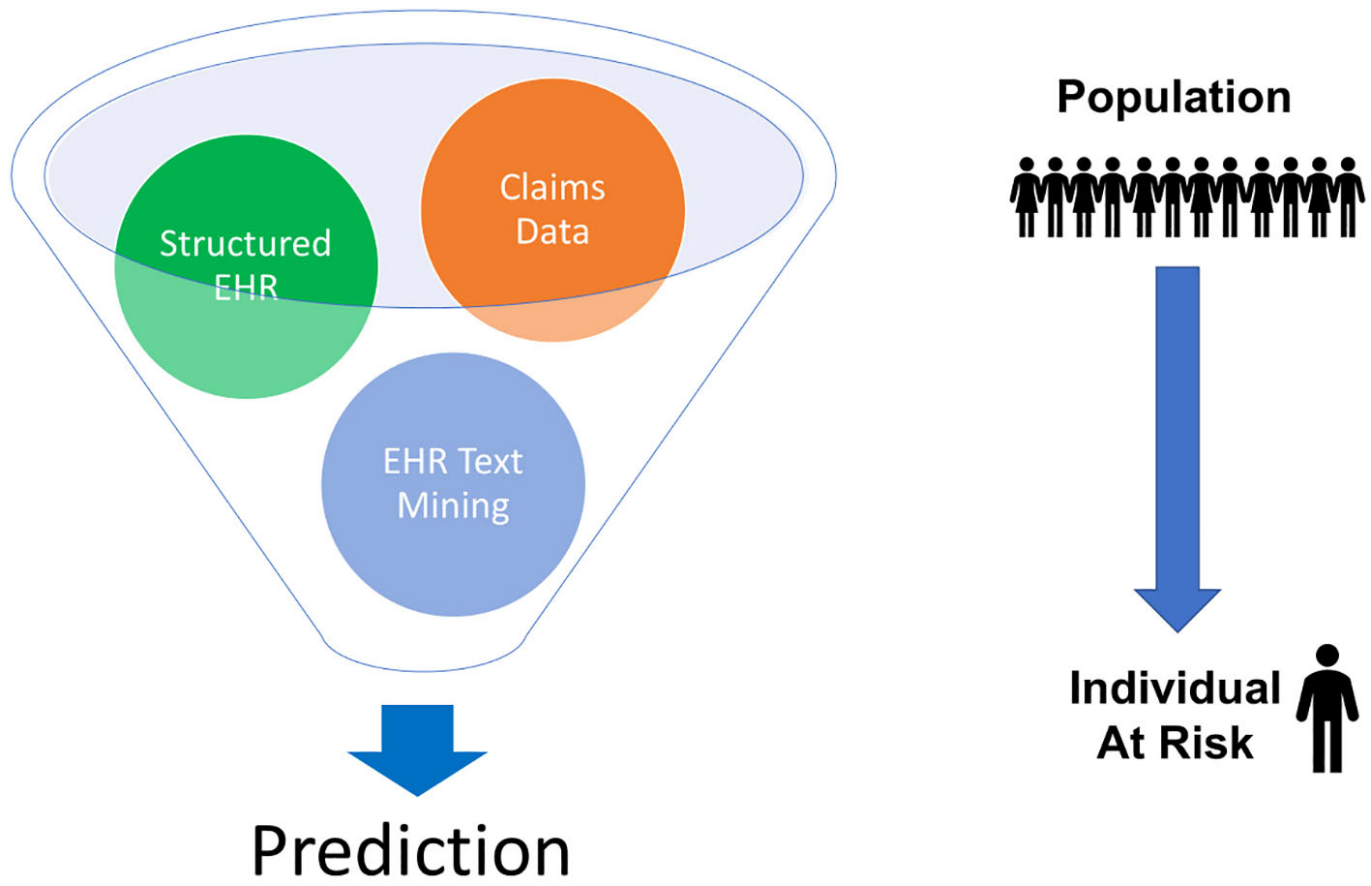
AI can use different types of structured and unstructured data to make predictions. For rare diseases, this can involve taking population data and using it to identify individuals at risk. EHR = electronic health records.

As PI patients make their journey through the healthcare system from primary care to local specialists or hospitals and potentially to PI-focused referral centers, the available data have shown how delayed diagnosis can adversely affect the patient’s well-being and better connections between local providers and referral centers could improve care. Looking towards the future and taking advantage of new technologies, the question becomes: how can a data-driven AI approach be used to improve care and enhance safety for patients with PI and across the healthcare spectrum? There is a lot of interest and some hype surrounding the use of AI and machine learning in healthcare. AI has been applied to a wide variety of settings and has shown some benefit in terms of predicting trends and outcomes (34). A strength of AI in making these types of predictions is that AI can make use of both structured and unstructured data (**Figure 4**) (35).

Probably one of the most well-known facets of AI and machine learning are the large language models (e.g. ChatGPT). For a generalized medical large language model, many different forms of data (images, electronic health records, patient monitor outputs, genomics, proteomics, and graphical data) can be used in a multimodal self-supervised training and combined with several medical knowledge domains (e.g., medical literature and clinical notes) (36). This will allow these systems to generate a number of applications: radiology reports that are grounded by specific identification of image characteristics, chatbots to address patients’ questions, interactive video augmentation of surgical procedures, bedside decision support that can provide predictive warnings and care recommendations and interactive note-taking that can lessen the administrative burden on providers (36).

**Figure 5:**
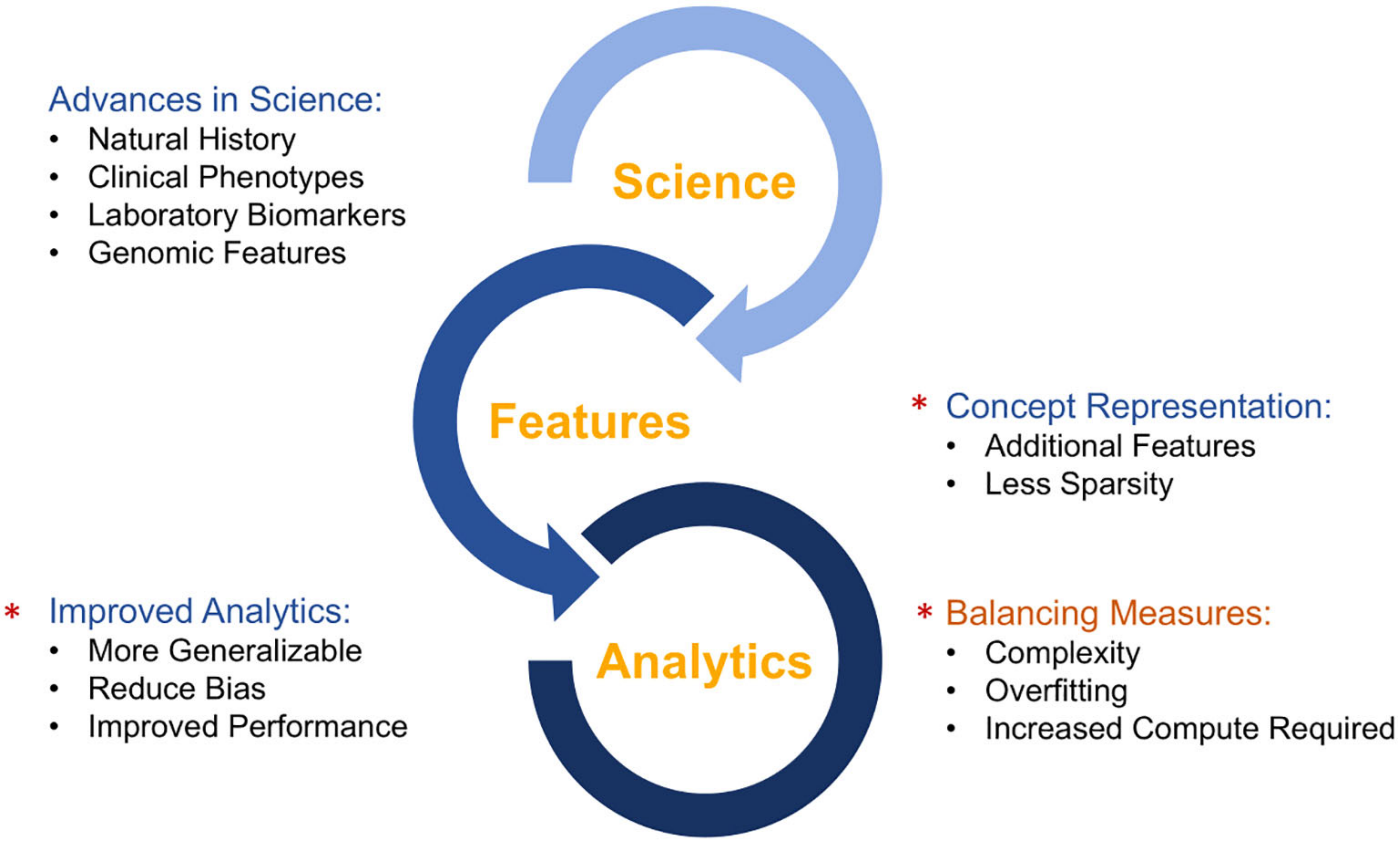
Synergy between developments in clinical science and artificial intelligence/machine learning.

PI is clearly a complex field with a tremendous amount of underlying data. One way of shortening the diagnostic delay is to apply AI, machine learning and data science to the vast pool of knowledge in PI (**Figure 5**). Using the existing and developing database of the natural history of PI, the clinical features can be selected that will serve as the basis for building new models. AI and machine learning can be used to improve the data analysis models to make them more applicable to all patients, reduce any inherent bias and improve its predictive performance. The approaches currently being taken to apply AI and machine learning to PI include the use of electronic health records, claims data, single nucleotide variants and flow cytometry data as features to feed into standard machine learning or neural networks (37–39). The goal of these models is to predict the risk of PI using phenotypic patterns, to identify PI patients based on flow cytometry patterns and to use records of prior treatment to predict the diagnosis of PI (37–39).

## 9 A data-driven approach

**Figure 6:**
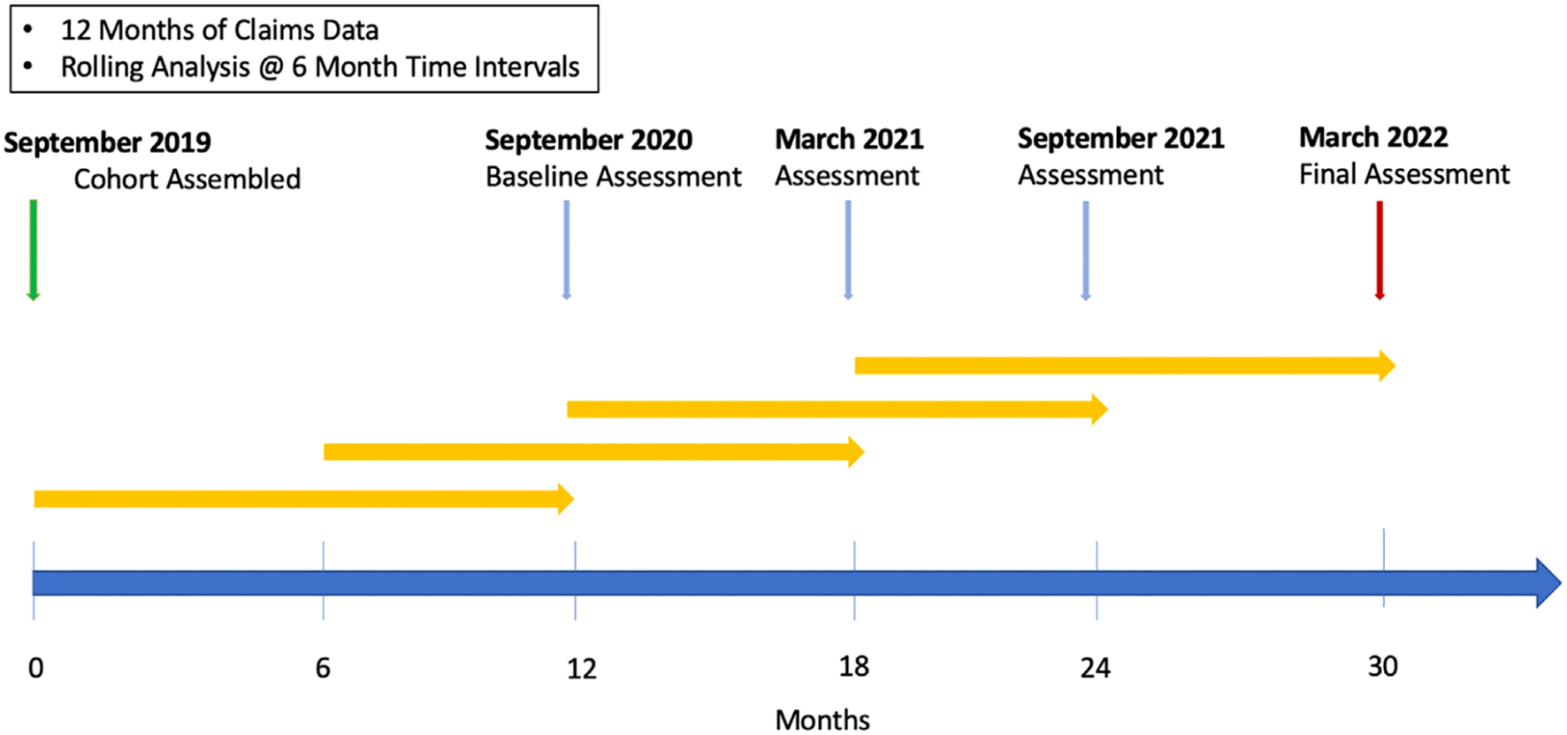
“AI for PI” Study timeline. In this prospective, three-year, longitudinal study at Texas Children’s hospital. Claims data from a large population (n = 427,110) was subject to rolling, periodic analysis over a period of 30 months. Reprinted from Rider et al. (22) with permission from Elsevier.

Our approach has been to use a multi-modal data approach. Combined claims data and structured electronic health records data were used as well as text mining of electronic health records. These data were brought through an ensemble of classifiers to make a prediction from population level input down to an individual who may be at risk of PI. This approach was applied in a three-year longitudinal Medicaid claims data study at a single center, Texas Children’s Hospital (**Figure 6**) (22).

At six-month intervals starting at 12 months, a year’s worth of claims data was analyzed using the SPIRIT analyzer from the Jeffrey Modell Foundation, a program for recognition and tracking of PI (23, 40). This program recognizes specific diagnosis codes, pharmacy codes and Current Procedural Terminology (CPT) codes with a prespecified weighting based on the 10 warning signs (20). This produces a risk score for each individual. Then at the end of the three-year period a retrospective analysis was completed to assess how many individuals designated low-risk were diagnosed with PI versus how many individuals designated high-risk were diagnosed with PI (22).

The demographics of this cohort were representative of the geographic area around the study center (equal sex distribution (53% female) and 46% Hispanic). This initial risk categorization by the SPIRIT Analyzer found that 0.07% of the cohort were classified as high-risk. In this high-risk group 9% were diagnosed with PI compared to 1.5% in the low-medium risk group and 0.2% in the group with no claims of interest (p < 0.001) (22).

A second analytical step was used to further stratify high-risk individuals and allow prioritization of further diagnostics through referrals and additional evaluations. This model was trained on 612 high-risk individuals and 287 patients with known IEI. Output from this model was passed into a model based on the number of healthcare visits over a 12-month period. Several different models were tested for their performance in this scenario and the models chosen were a dense neural network with one layer containing 128 nodes and a regularized (L1) logistic regression classifier. These machine learning models filtered the high-risk group into those that were referred to a clinical immunologist and those that would be continually followed and evaluated (22).

Out of the population of 427,110, there were 37 individuals that were deemed appropriate for referral by the model and manual chart review (**Table 3**). The number of expert-defined appropriate referrals was the study primary endpoint. Of these 37 patients, 16 were available for follow-up at the study center. While these patients were not followed post referral, and out of study scope, those referred were expected to have immunologic disease. When the PI risk ratio of patients diagnosed with PI in the selected for referral group (n = 7; 0.34 ± 0.24) was compared to the PI risk ratio for the high-risk population not selected for referral (n = 329; 0.27 ± 0.18) for the September 2021 and March 2022 analyses, the difference was statistically significant (p < 0.001). The end result is a two-step model that can help determine which individuals in a population should be referred to a clinical immunologist for further evaluation (22).

**Table 3:** Triage outcomes for the Texas Children’s Health Plan cohort in the “AI for PI” study.

Analysis Date	Cohort Size (n)	Triage Assessment (n)	Referral Candidates (n)	Referrals
March 2021	427,110	Possible PI 160 Under Eval 11 Known PI 5 Unlikely 114	30	12
September 2021	337,609	Possible PI 278 Under Eval 7 Known PI 7 Unlikely 260	4	2
March 2022	320,619	Possible PI 52 Under Eval 2 Known PI 7 Unlikely 43	3	2
Total			37	16

Reprinted from Rider et al. (22) with permission from Elsevier.

Another data-based AI approach is to assess the utility of the previously described 10 Warning Signs of PI (20). Starting with a database of approximately 152 million EHR, a cohort of patients with verified IEI was identified through two instances of a verifiable IEI diagnosis in their EHR (n = 41,080). This cohort was matched to a cohort of 250,262 controls (24). The 10 warning signs were encoded along with their weighting factors and relevant diagnosis codes. The number of individuals with >2 warning signs was calculated and compared between the IEI and control cohorts. In addition, the dataset was used to estimate the prevalence of IEI in the US population and to assess which warning signs were most heavily weighted in this cohort.

The proportion of patients with >2 warning signs was significantly higher in the IEI group (0.33) than in the controls (0.031, p < 0.0005). The number of IEI patients in the data set (41,080) was derived from a data set of 73,165,655 EHR from patients < 61 years old with at least one year of data. This corresponds to a prevalence of 0.056% or approximately 6 cases of IEI in 10,000 individuals. When applied to the current US population < 61 years old, this results in an estimate of 154,820 individuals < 61 years old that are affected with IEI in the US (24).

**Figure 7:**
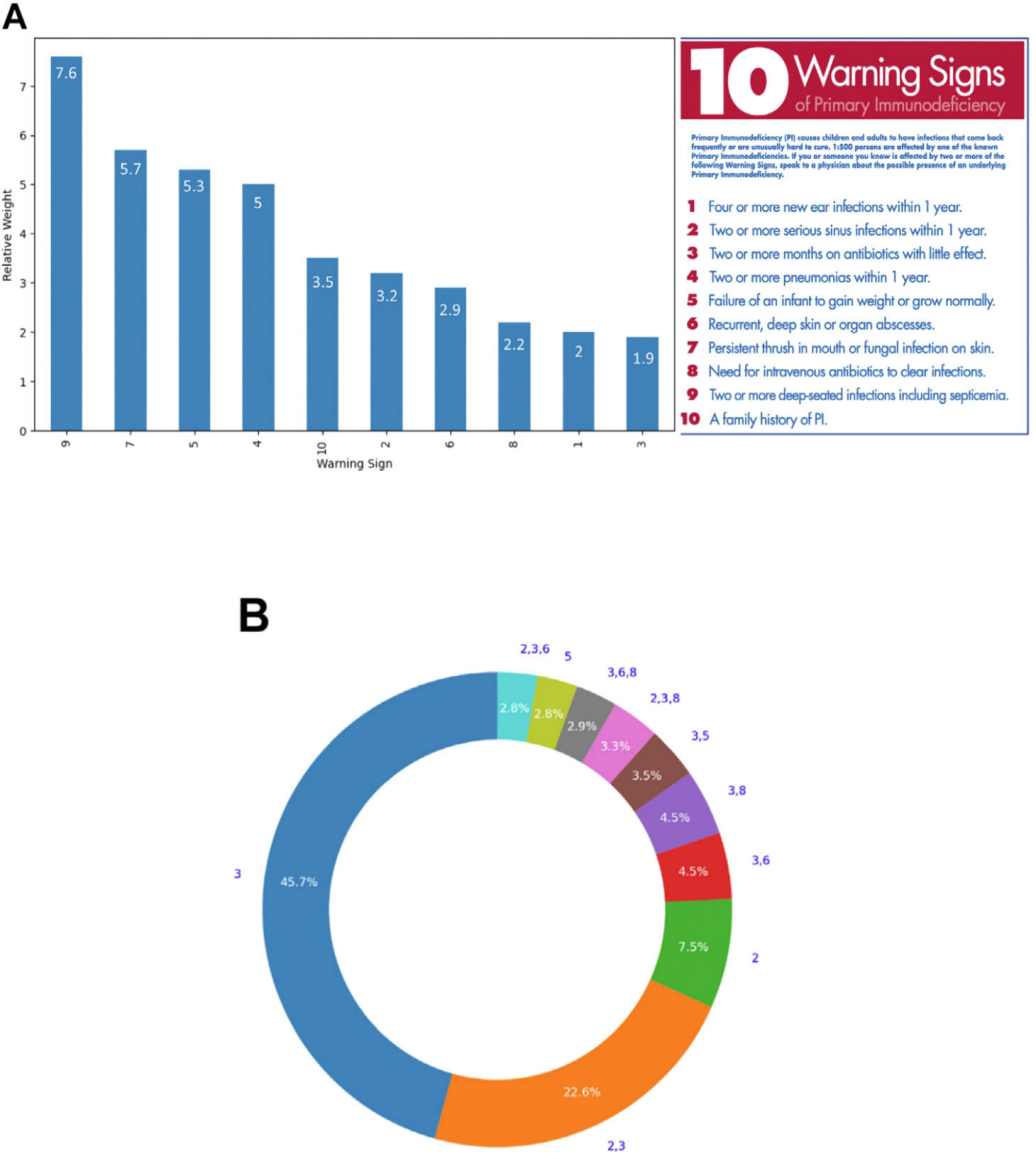
**(A)** The relative weight of each of the 10 Warning Signs of PI. The relative weight is the ratio of the Warning Sign per patient in PI patients to the Warning Sign per patient in controls. These warning signs were developed by the Jeffrey Modell Foundation Medical Advisory Board (20). Consultation with Primary Immunodeficiency experts is strongly suggested. © 2024 Jeffrey Modell Foundation. **(B)** The 10 most common warning sign patterns in IEI patients that showed at least one warning sign. The numbers outside the ring are the warning sign numbers and the numbers in the ring are the percentage for that pattern in the top 10 patterns. Reprinted from Rider et al. (24) with permission from Elsevier.

The presence of the 10 warning signs was evaluated in patients with IEI and controls. The ratio of patients with each warning sign to the total number of IEI or control patients was calculated and compared between the groups (**Figure 7**). For example, the most heavily weighted warning sign, #9 (Two or more deep-seated infections including septicemia) was 7.6-fold more common in the IEI patients than in controls. Other heavily weighted Warning Signs included #7 – Persistent thrush in mouth or fungal infection on skin, #5 – Failure of an infant to gain weight or grow normally, and #4 – Two or more pneumonias within one year.

**Figure 7B** shows the top 10 warning sign patterns in the IEI patients studied with at least one warning sign (n= 14,900). The most common warning sign patterns were warning sign #3 (Two or more months on antibiotics with little effect) and warnings signs #2 (Two or more serious sinus infections within one year)- and #3.

These findings suggest that the original 10 warning signs (20) continue to have value in the diagnosis of IEI. Weighting of the warning signs and assessment of their accumulation over time may be useful in improving the identification and monitoring of patients that are at high risk of IEI and hastening their diagnosis and treatment.

**Figure 8:**
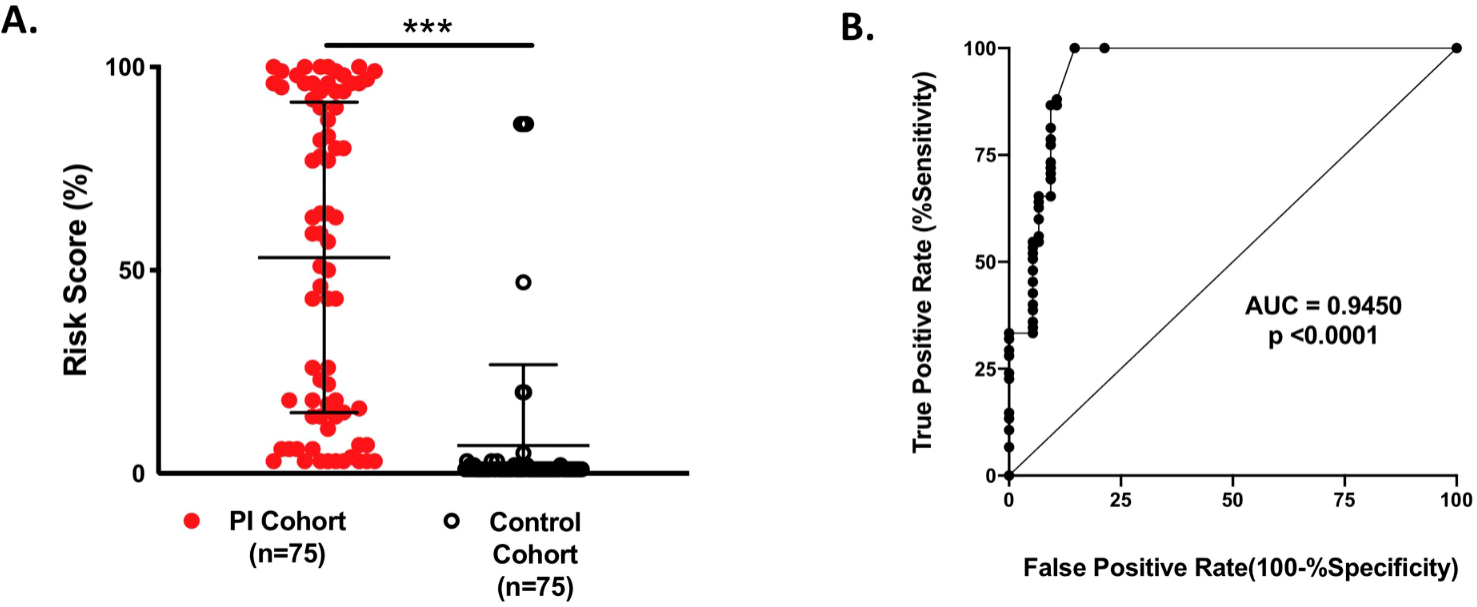
**(A)** Testing the validity of the Bayesian network model for 150 individual patients: 75 PI patients and 75 controls. The risk scores were significantly different between the groups: 53% PI cohort and 7% control cohort; ***p < 0.000001. **(B)** Area under the receiver operator characteristic curve (AUROC) was used to evaluate the performance of the model. From Rider et al. (41) licensed under CC-BY.

In another approach, a Bayesian network was used to demonstrate that PI concepts can be derived from existing data, specifically mined from EHR, used in a machine learning model to provide diagnostic guidance and assessment of disease risk (41). After training the model using cohorts of known PI patients and controls, it was validated by comparing the risk scores of the PI cohorts with those of the control cohort (n = 75 for each group). As shown in **Figure 8A**, the mean risk scores for the two populations were significantly different (p < 0.000001) with a risk score of 53% for the PI cohort and 7% for the control cohort. When the performance of this model was assessed using the area under the receiver operator characteristic curve (AUROC) (**Figure 8B**, the AUC was 0.9450. A value of 1 for AUC indicates that the model detects the difference between the cohorts 100% of the time (41).

## 10 Conclusions

Each type of model (expert-driven and data-driven) has theoretical advantages and disadvantages. The expert-driven model has as its primary advantages that it takes advantage of human expertise in rare diseases and can utilize unpublished data. One of the primary drawbacks of this approach is that it is time-consuming. Data-driven models have the advantages of reproducibility, the ease of application of AI tools and faster results but also suffer from the potential disadvantages of vulnerability of the results due to poor quality or inaccurate data.

The ultimate goal of both the expert-driven and data-driven approaches is to create models that are not cloistered in a laboratory but to create models that are useful and widely available tools. To utilize the wealth of data and clinical expertise available in a structured way to create models that support clinical decisions and potentially improve diagnosis and treatment. In the case of PI, this could be reflected in earlier diagnosis and treatment that would improve the health and quality of life for these patients. Through earlier diagnosis and treatment, the potential for permanent damage from repeated infections could be reduced and some of the non-infectious complications of these diseases might be avoided.

As PI patients make their journey from the onset of symptoms to diagnosis and treatment, healthcare providers have several tools to help shorten the time from onset to diagnosis and avoid the negative sequalae of a delayed diagnosis. The new approaches described herein aim to utilize new existing and tools in novel ways to enhance diagnostic accuracy and enhance connections between different treatment centers to provide improved care for PI patients. Additional studies are needed to refine these tools, expand their use and to potentially combine them to provide the most efficient and effective care to patients with PI.
